# Homoploid hybrid origin of *Yucca gloriosa*: intersectional hybrid speciation in *Yucca* (Agavoideae, Asparagaceae)

**DOI:** 10.1002/ece3.328

**Published:** 2012-08-01

**Authors:** Jeremy D Rentsch, Jim Leebens-Mack

**Affiliations:** Department of Plant Biology, University of GeorgiaAthens, Georgia, 30602

**Keywords:** Hybrid index, hybrid speciation, microsatellite analysis, *Yucca*

## Abstract

There is a growing appreciation for the importance of hybrid speciation in angiosperm evolution. Here, we show that *Yucca gloriosa* (Asparagaceae: Agavoideae) is the product of intersectional hybridization between *Y. aloifolia* and *Y. filamentosa*. These species, all named by Carl Linnaeus, exist in sympatry along the southeastern Atlantic coast of the United States. *Yucca gloriosa* was found to share a chloroplast haplotype with *Y. aloifolia* in all populations sampled. In contrast, nuclear gene-based microsatellite markers in *Y. gloriosa* are shared with both parents. The hybrid origin of *Y. gloriosa* is supported by multilocus analyses of the nuclear microsatellite markers including principal coordinates analysis (PCO), maximum-likelihood hybrid index scoring (HINDEX), and Bayesian cluster analysis (STRUCTURE). The putative parental species share only one allele at a single locus, suggesting there is little to no introgressive gene flow occurring between these species and *Y. gloriosa*. At the same time, diagnostic markers are segregating in *Y. gloriosa* populations. Lack of variation in the chloroplast of *Y. aloifolia*, the putative maternal parent, makes it difficult to rule out multiple hybrid origins of *Y. gloriosa*, but allelic variation at nuclear loci can be explained by a single hybrid origin of *Y. gloriosa*. Overall, these data provide strong support for the homoploid hybrid origin of *Y. gloriosa*.

## Introduction

Interspecific hybridization is known to be an important evolutionary process contributing both to genetic variation within species and to the origin of new species, especially in plants (Anderson 1949; Grant 1958, 1981; Stebbins 1959; Arnold 1992). Hybrid speciation may involve allopolyploidization followed by diploidization through fractionation (Schnable et al. 2011; Severin et al. 2011) or admixture and recombination of two parental genomes without change in ploidy (Rieseberg 1991; Arnold 1993; Ren et al. 2012; Brennan et al. 2012). The genomes of both allopolyploid and homoploid hybrid species are typically mosaics of their parental genomes (but see Jiggins et al. 2008), but whereas polyploid hybrid species acquire the full chromosomal sets from both parents, homoploid hybrids meld chromosomal segments from both parents while remaining diploid. The processes that give rise to polyploid versus homoploid hybrid species do not appear to be random. Recent reviews have shown that the parents of homoploid hybrid species are typically more genetically similar to each other than the parents of allopolyploids (Chapman and Burke 2007; Paun et al. 2009).

Chromosome doubling in polyploid hybrids creates an instant barrier to reproduction with the parental species (Ramsey and Schemske 1998). However, because homoploid hybrids retain the chromosome count of their parental species, barriers to reproduction with parental species may remain more porous and speciation may be less likely. Homoploid hybrid speciation is hypothesized to involve reproductive isolation between parent and hybrid populations due to resorting of chromosomal segments and traits from both parents to produce a unique constellation of traits in the hybrid species (Müntzing 1930; Stebbins 1957; Grant 1958; Rieseberg 1997). However, homoploid hybrid speciation may be driven by introgression or transgressive segregation of a single locus as has been shown in Heliconius butterflies (Salazar et al. 2008; The Heliconius Genome Consortium 2012). Both scenarios are consistent with the hypothesis that a novel trait or suite of traits in the hybrid species can promote ecological isolation between hybrid and parental populations (Buerkle et al. 2000; Rieseberg et al. 2003; Gross and Rieseberg 2005).

As predicted, most documented examples of homoploid hybrid speciation involve some form of ecological divergence between parental species and their hybrid progeny. Examples include habitat divergence in *Iris, Helianthus*, and *Pinus* (Rieseberg 1991; Arnold 1993; Wang et al. 2001), pollinator divergence in *Penstemon* (Wolfe et al. 1998), and the divergence of multiple ecological factors in the genus *Hyobanche* (Wolfe and Randle 2001). It is also possible for hybrids to form in sympatry but only become reproductively isolated from parental species in allopatry as demonstrated in *Senecio* (James and Abbott 2005).

The genus *Yucca* contains approximately 40 species with most diversity found in Mexico and the southwestern United States. Two monophyletic sections include most of the species within the genus (Pellmyr et al. 2007; Smith et al. #b^508^): Chaenocarpa with capsular-fruited yuccas and *Yucca* (syn, Sarcocarpa) with fleshy-fruited species. A third clade, Clistocarpa, includes only *Yucca brevifolia* (Joshua tree) with two described varieties (Smith et al. 2008b, 2009). All *Yucca* species share a fascinating mutualistic relationship with pollinating yucca moths within the genera *Tegeticula* and *Parategeticula* (e.g., Trelease 1902; Pellmyr #b^502^). Female yucca moths actively gather pollen from yucca anthers and insert the pollen into the yucca's cup-shaped stigmatic surface after inserting eggs into the carpel or style of the flower. Developing moth larvae then feed on yucca seeds. The majority of seed-feeding insects involved in plant pollination mutualisms display high host specificity (Fleming and Holland 1998; Weiblen 2002; Kato et al. 2003; Pellmyr and Seagraves 2003). Yucca–yucca moth associations generally exhibit narrow specificity with 60% of pollinating moths visiting a single host (Pellmyr #b^503^, #b^502^). The most significant departure from this pattern is the broad host range exhibited by the pollinating moth *Tegeticula yuccasella*, which utilizes seven host species (Althoff et al. 2012).

It is thought that pollinator specificity may discourage interspecific hybridization through highly correlated plant and pollinator phenotypes. In the fig–fig wasp pollination mutualism, the wasp's ovipositor length is significantly correlated with the length of the fig's flora style (Weiblen 2004). Similarly, unpublished data (Pellmyr and collaborators) from the Yucca–yucca moth system suggest there is a significant correlation between the length of the yucca moth's ovipositor and the thickness of the yucca's carpel. A cross pollination event in which phenotypes do not match could lead to increased mortality for pollinator eggs and early instars. Nevertheless, hybridization has been documented between *Y. baccata* and *Y. schidigera* (Hanson 1992; Leebens-Mack et al. 1998), *Y. baccata* and *Y. torreyi* (Miles 1983), and between *Y. brevifolia var. brevifolia* and *Y. brevifolia var. jaegeriana* (Smith et al. 2009). These hybridization events likely result from pollen transfer between a moth's typical host and a sympatric Yucca species that is typically pollinated by another moth species. Although hybridization appears to be more common within distinct sections of the genus, it is certainly possible that the phenomenon is widespread, even occurring between plants in different sections. Morphological evidence from yuccas sampled in the Four Corners Region of the United States (Arizona, Colorado, New Mexico, and Utah) suggests that the fleshy-fruited species *Y. baccata* and *Y. madrensis* may hybridize with the capsular-fruited *Y. elata* to produce intersectional hybrids, although few individuals were described (Lenz and Hanson #b^501^). Sympatric *Yucca* species pollinated by *T. yuccasella* in the southeastern United States may provide the best opportunity to detect and characterize intersectional hybridization within the genus.

Here, we test the hypothesis that *Y. gloriosa* is the product of intersectional hybridization between *Y. aloifolia* (section: Yucca) and *Y. filamentosa* (section: *Chaenocarpa*). These three diploid species (Bonnet 1912; Watkins 1936) occur sympatrically along the southeastern Atlantic coast of the United States (Brown 1959) and share *T. yuccasella* as a pollinator (Pellmyr and Leebens-Mack 1999; Leebens-Mack and Pellmyr 2004), although *Y. aloifolia* might also be pollinated by nonmoth visitors as well (Engelmann 1873; Riley 1891). *Yucca aloifolia* is thought to be a relatively recent addition to the flora of the southeastern United States possibly as a consequence of both human-mediated dispersal (Gerarde 1633; Dunbar 1958) and natural dispersal (Trelease 1902). Furthermore, the species are known to partially overlap in their flowering phenology across much of their range (Groman and Pellmyr 2000). William Trelease (1902) suggested that *Y. gloriosa* exhibited a blend of *Y. aloifolia* and *Y. filamentosa* traits and hypothesized that *Y. gloriosa* was a hybrid likely limited to vegetative propagation. While hybrid species are not always morphologically intermediate, *Y. gloriosa* displays a fruit type that appears to be intermediate to the capsular and fleshy fruits of yuccas in sections Chaenocarpa and Yucca, respectively. In this study, we use a combination of nuclear microsatellite data and chloroplast sequence data to address the following questions: (1) is *Y. gloriosa* the product of intersectional hybridization within *Yucca*, (2) is there evidence for sexual reproduction within *Y. gloriosa* populations, (3) is there a signature of introgressive gene flow between *Y. gloriosa* and either parental species (*Y. aloifolia* or *Y. filamentosa*), and (4) are the marker data consistent with a single origin or multiple origins of the hybrid species *Y. gloriosa*.

## Materials and Methods

### Plant material collection and DNA extraction

**Figure 1:**
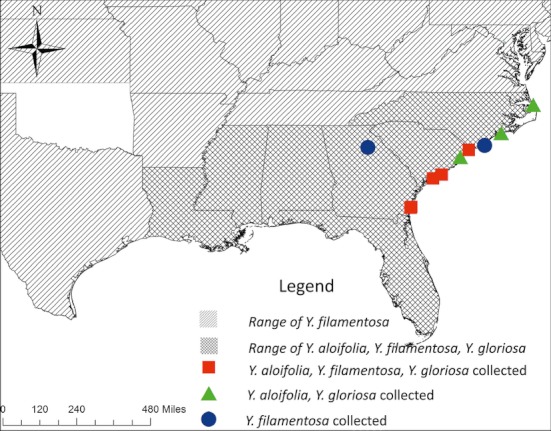
Location of field sites, species collected per site, and overall range of *Yucca aloifolia*, *Y. filamentosa*, and *Y. gloriosa* in the United States.

Leaf material was collected from seven populations of *Y. aloifolia* (*n* = 32), six populations of *Y. filamentosa* (*n* = 29), and seven populations of *Y. gloriosa* (*n* = 35) primarily along the southeastern coast of United States (Fig. 1). While these species are distributed across the southeastern United States, they are only found reliably in sympatry along the Atlantic coast. Approximately 1 g of leaf material was harvested from each sample for DNA extraction. Material was flash frozen in liquid nitrogen until it could be stored in the laboratory at −80°C. Whole genomic DNA was extracted using a modified cetyltrimethylammonium bromide protocol (Doyle and Doyle 1990). Several voucher specimens were collected from each population and deposited in the University of Georgia herbarium (GA).

### Chloroplast haplotype analysis

Chloroplast markers were developed by aligning the *Y. filamentosa* and Hosta chloroplast genomes (McKain et al. unpubl. ms.) and identifying the most variable regions between the two. The following markers were amplified and sequenced for six individuals per species in order to identify loci with interspecific polymorphisms: atpF-atpL, petA-psbJ, rpl20-rps12, tabE-F, trnT-trnL, ndhC-trnV, and ycf4-cemA. Polymearse chain reactions (PCRs) were performed in 20 *μ*L volumes containing 1.5 *μ*L of template DNA (approximately 10 ng), 17.0 *μ*L sterile distilled water, 2.5 *μ*L tricine taq buffer (0.37 mmol/L tricine, and 0.61 mmol/L KCL), 1.5 *μ*L of 25 mmol/L MgCl2, 0.5 *μ*L dNTP mixture (containing equal parts: 2 mmol/L dATPs, 2 mmol/L dCTPs, 2 mmol/L dGTPs, and 2 mmol/L dTTPs), 1.0 *μ*L of 10.0 *μ*mol/L reverse primer, 1.0 *μ*L of 10.0 *μ*mol/L forward primer, and one unit of taq polymerase. Cycling conditions were as follows: initial denaturation at 95°C for 2 min; 35 cycles of 94°C for 30 sec, 54°C for 30 sec, and 72°C for 90 sec, followed by a final extension at 72°C for 5 min. PCR products were purified by incubation with Exonuclease I and Shrimp Alkaline Phosphatase at 37°C for 15 min, followed by a 15-min enzyme inactivation step at 70°C. PCR products were then sequenced in separate reactions for the forward and reverse primers using BigDye® Terminator v3.1 chemistry. Reactions conditions largely followed the manufacturer's protocols; however, approximately one-third of the suggested amount of BigDye® was used per reaction. Unincorporated ddNTPs were removed using Sephadex, a cross-linked dextran gel. Sanger sequencing was performed at the Georgia Genomics Facility (GGF) on an Applied Biosystems (Carlsbad, CA) 3730xl 96-capillary DNA Analyzer.

### Microsatellite development and genotyping

A transcriptome assembly for *Y. filamentosa* (OneKP consortium, unpubl. data; http://www.onekp.com) was scanned for microsatellite repeats using MSATCOMMANDER (Faircloth 2008). MSATCOMMANDER identifies simple repeats and uses Primer3 (Rozen and Skaletsky 2000) to design flanking PCR primers. Primer pairs were tested for amplification in both hypothesized parental species. Three individuals per species were selected for initial genotyping in order to detect interspecific variation in microsatellite repeat number. Ultimately, 14 of 55 screened loci were selected based on their polymorphic nature and ability to amplify reliably in all three species (Table 1).

**Table 1:** Microsatellite loci found to be variable between *Yucca aloifolia* and *Y. filamentosa*

	Microsatellite	Repeat motif	Size range
MSATYF01-Forward	CCGACTTCCACCGAACTTG	(CAG)∧5	181–201
MSATYF01-Reverse	AGACCCAGCGATGATGGAG		
MSATYF03-Forward	TCAAAAGCCCCAAGAACCC	(CAT)∧4	180–201
MSATYF03-Reverse	CGATTCTCTGACCGGCGTG		
MSATYF04-Forward	TCTTCCTCTGCCCAAAGCC	(CAA)∧2 (CCT)∧5	195–201
MSATYF04-Reverse	TGCAGCTTCCTTGGAACAC		
MSATYF12-Forward	AATGCAAGCCCTCCTCCTC	(CTT)∧4	180–186
MSATYF12-Reverse	GGGTTTTCCTTGGCACACG		
MSATYF13-Forward	TTACCGAAGCCAGCTCTGC	(AG)∧6	234–243
MSATYF13-Reverse	GGAGTGAGAGAGGGAGTGG		
MSATYF16-Forward	TGATTCCTGAACCCAGCCC	(CCG)∧4	165–171
MSATYF16-Reverse	GGGGTGATGGAGTAGGCAC		
MSATYF28-Forward	CATGGCCACAGCCATTGAG	(AAG)∧4	231–256
MSATYF28-Reverse	CACAAATCGAGCTCCAGCG		
MSATYF30-Forward	CCACCATTCCGTCACACTC	(CCG)∧4	247–251
MSATYF30-Reverse	CCATGCGGCGTTCTTGATG		
MSATYF41-Forward	ACACTCCAGTCCTGCATCC	(TGA)∧4	239–247
MSATYF41-Reverse	AAGTGATCCAACATGAACATCC		
MSATYF43-Forward	ACAGCAATAAGCAGGAGATAGG	(TTC)∧5	261–279
MSATYF43-Reverse	GGCCTTTTGGCTTCTGCTC		
MSATYF44-Forward	TTCGAGCAGCCAGAGGAAC	(GCC)∧4	257–266
MSATYF44-Reverse	ACGCCAAGGAGAAGGACAG		
MSATYF51-Forward	GTTCTCTGTCAAATTGGTTGCG	(CAT)∧5	256–272
MSATYF51-Reverse	TGCTGTGTGGTGACTTGTG		
MSATYF52-Forward	TCTCAGTCTCGATGGACCC	(GCC)∧4	175–181
MSATYF52-Reverse	GGTCTTTGTCAGGAACGGC		
MSATYF53-Forward	CGATCAACTGTGACATCCGC	(CTG)∧5	322–328
MSATYF53-Reverse	CTAGTCGTCCTGCACTCCC		

A three primer PCR protocol was utilized to fluorescently label PCR products using a universal M13(-21) primer (Schuelke 2000). Reactions were performed in 15 *μ*L volumes containing 1.5 *μ*L of template DNA (approximately 10 ng), 7.5 *μ*L sterile distilled water, 3.6 *μ*L tricine taq buffer (containing 0.02 mmol/L MgCl2), 0.37 mmol/L tricine, and 0.61 mmol/L KCL), 0.06 *μ*L dNTP mixture (containing equal parts: 2 mmol/L dATPs, 2 mmol/L dCTPs, 2 mmol/L dGTPs, and 2 mmol/L dTTPs), 0.4 *μ*L of 10.0 *μ*mol/L reverse primer, 0.4 *μ*L of 10.0 *μ*mol/L M13(-21) primer, 1.0 *μ*L of 1.0 *μ*mol/L forward primer, and one unit of taq polymerase. Thermocycle conditions followed a touchdown protocol as follows: initial denaturation 94°C for 5 min; 10 cycles of 94°C for 30 sec, 63°C for 30 sec with a 1°C drop each cycle, and 72°C for 30 sec; 27 cycles of 94°C for 30 sec, 56°C for 30 sec, and 72°C for 1 min; followed by a final extension at 72°C for 5 min. Products were diluted 1:15. A mixture of Rox dye-labeled size standard and formamide (in a 1:10 ratio) was added to each sample. Fragment analysis was performed on an Applied Biosystems 3730xl DNA Analyzer.

### Data analysis

Chloroplast sequence data were assembled and inspected using Sequencher® version 4.7. Nuclear microsatellite genotype data were visualized and scored using ABI's Peak Scanner™ software. The uncorrected p distance between *Y. aloifolia* and *Y. filamentosa* was calculated from a combined data set utilizing six samples per species and all seven sequenced chloroplast loci. Nucleotide alignments were made using MUSCLE (Edgar 2004), and the uncorrected p distance of the combined data set was calculated in Mesquite (Maddison and Maddison 2007)

Multilocus nuclear microsatellite data were displayed graphically using principal coordinate analysis (PCO) as incorporated into GenAlEx version 6.41 (Peakall and Smouse 2006). This analysis utilizes a covariance matrix based on genetic distance to plot individuals based on the variance among their multilocus genotypes.

The hypothesis that *Y. gloriosa* is a homoploid hybrid species was first tested through assessment of admixture using STRUCTURE (Pritchard et al. 2000; Falush et al. 2003). STRUCTURE uses a Bayesian clustering algorithm to probabilistically assign the proportion of ancestry of unknown individuals into one or more source populations. In order to determine the appropriate number of clusters given the data, all individuals were included in initial analyses without a priori species designation. These data were analyzed for K values ranging from one to nine with five replicates per K. Each run had an initial burn-in period of 50,000 iterations, followed by 500,000 Markov chain Monte Carlo iterations. The ad hoc statistic ΔK (Evanno et al. 2005), as calculated by STRUCTURE HARVESTER (Earl and vonHoldt 2011), was used to verify the separation of the parental species into distinct clusters.

Following the methods of James and Abbott (2005), STRUCTURE was next used to approximate the proportion of the hybrid's nuclear genome that was inherited from each hypothesized parental species. Each parental species (*Y. aloifolia* and *Y. filamentosa*) was set as a distinct population, while the hybrid individuals (*Y. gloriosa*) were treated as having an unknown ancestry. In order to utilize the “learning samples” function, USEPOPINFO was invoked, allowing for the data from individuals with a known ancestry to help inform the classification of individuals with an unknown ancestry. As before, each of five runs had an initial burn-in period of 50,000 iterations, followed by 500,000 Markov chain Monte Carlo iterations. All five runs were assessed for convergence.

The allelic composition of the putative hybrid's nuclear DNA was also investigated using HINDEX (Buerkle 2005), a maximum-likelihood estimator of hybrid index scores. HINDEX uses codominant marker data to estimate the proportion of alleles that were inherited from each parental species. Each *Y. gloriosa* individual was given a hybrid index score ranging from 0 to 1, representing individuals that were more *Y. filamentosa*-like and more *Y. aloifolia*-like, respectively. The likelihood function was determined by the frequency of each allele within the parental populations and by the unknown individual's genotype. For each multilocus genotype, the parent of origin was assigned for each locus using the approach of Gross et al. (2007).

## Results

### Chloroplast data

Of the seven chloroplast loci (a total of 11.4 kilobases) screened, only ndhC-trnV and trnT-trnL were variable between *Y. aloifolia* and *Y. filamentosa*. At the ndhC-trnV locus, the *Y. aloifolia* haplotype differed from the *Y. filamentosa* haplotype by a transition, a transversion, a 22-based pair insertion/deletion, and a mononucleotide microsatellite repeat. At the trnT-trnL locus, the *Y. aloifolia* haplotype differed from the *Y. filamentosa* haplotype only by a mononucleotide microsatellite repeat. These genomic changes between parental species resulted in an uncorrected p distance of 1.776 × 10^−4^. *Yucca aloifolia* and *Y. gloriosa* shared identical chloroplast haplotypes across all individuals and both loci.

### Nuclear data

Of the 55 putative microsatellite amplifying primer pairs screened, 14 (25.4%) were selected after verifying that they amplified a single locus exhibiting polymorphisms between *Y. aloifolia* and *Y. filamentosa*. Sixteen (32.7%) primer pairs amplified multiple loci in at least one species, 6 (10.9%) primer pairs produced null alleles in *Y. aloifolia*, while the remaining 19 (34.5%) primer pairs were monomorphic between species. Based on data from the 14 suitable loci, *Y. aloifolia*, *Y. filamentosa*, and *Y. gloriosa* had an average of 1.1, 2.6, and 1.8 alleles per locus, respectively. All *Y. aloifolia* samples were found have an identical multilocus genotype across all seven populations sampled.

**Figure 2:**
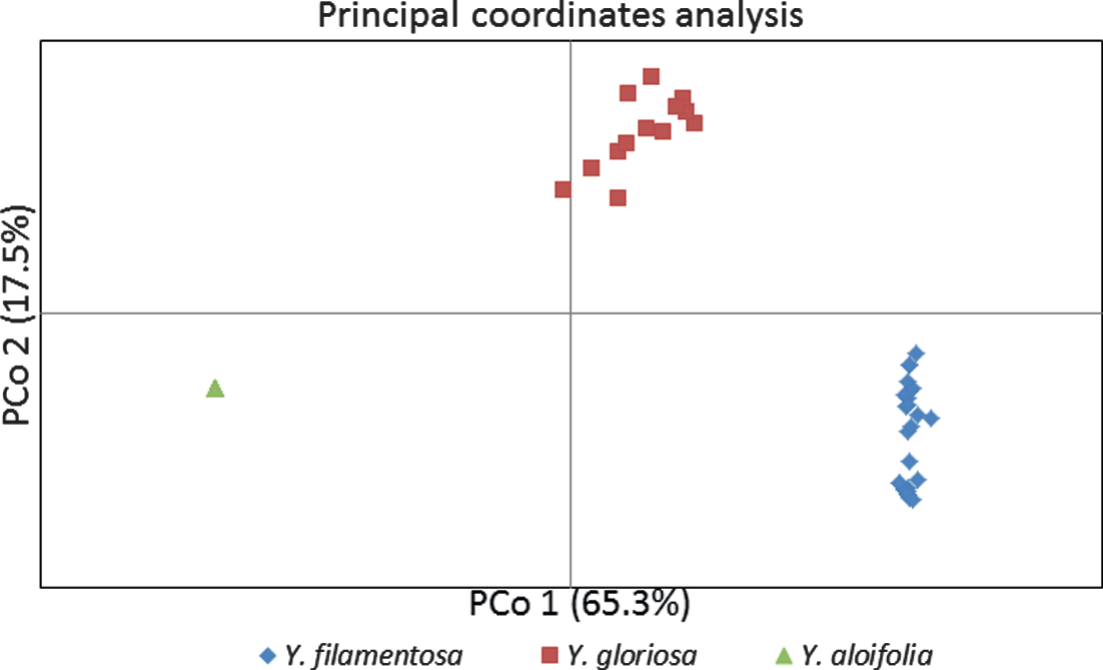
The first two axes of a principal coordinates analysis show distinct clusters of *Yucca aloifolia*, *Y. gloriosa*, and *Y. filamentosa* individuals found along the southeastern coast of the United States.

The PCO revealed three distinct clusters representing each of the species examined (Fig. 2). Along the first principal coordinate, which explains 65.3% of the variation between individual multilocus genotypes, *Y.gloriosa* appears to be intermediate between both hypothesized parental species. The clear separation of species into distinct clusters provides evidence of reproductive isolation between the parents and the putative hybrid. Backcrossed individuals would be expected to cluster much more closely to the parent with which they backcrossed.

**Figure 3:**
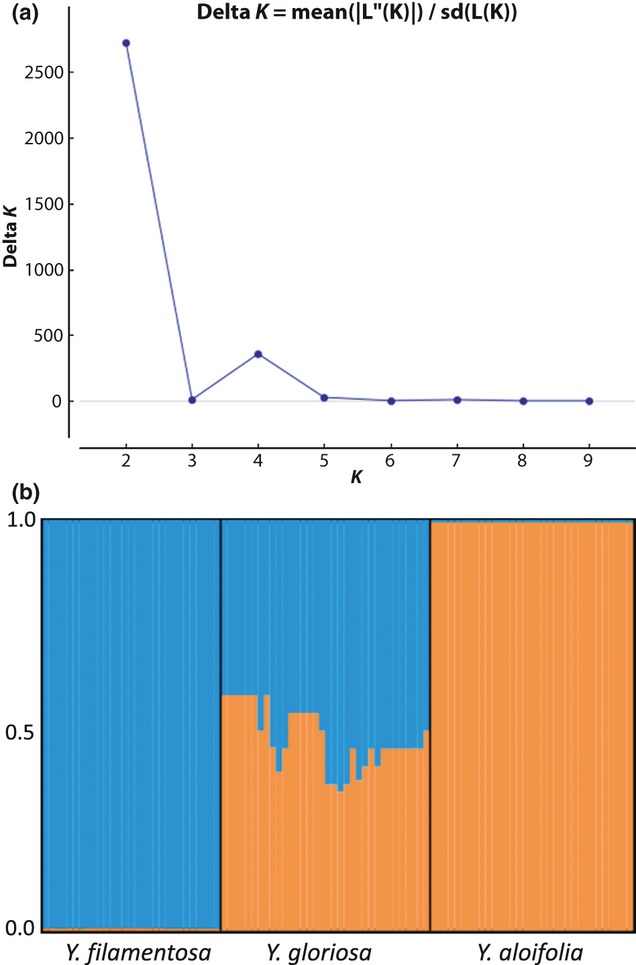
STRUCTURE analysis of *Yucca aloifolia*, *Y. filamentosa*, and *Y. gloriosa* multilocus genotypes. (a) Optimal number of clusters for the complete data set of nuclear microsatellite loci as calculated using the methods described by Evanno et al. and displayed graphically using Structure Harvester (Earl and vonHoldt 2011). (b) Estimated proportion of *Y. aloifolia* (orange) and *Y. filamentosa* (blue) nuclear alleles found in all individuals sampled from the southeastern coast of the United States.

**Figure 4:**
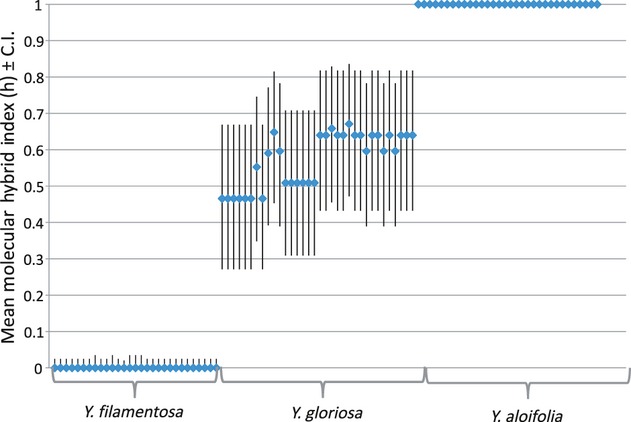
Maximum-likelihood estimates of molecular hybrid indices (±CI) based on 14 nuclear microsatellite loci for *Yucca gloriosa* and its putative parents, *Y. filamentosa* and *Y. aloifolia*. The hybrid index ranges from a score of 0 to 1, where 0 is completely *Y. filamentosa*-like and 1 is completely *Y. aloifolia*-like

Consistent with the hypothesis that *Y. gloriosa* is a product of intersectional hybridization between *Y. aloifolia* and *Y. filamentosa*, the methods of Evanno et al. (2005) identified two as the optimal number of clusters in the preliminary STRUCTURE analysis (Fig. 3a). In this analysis, *Y. aloifolia* and *Y. filamentosa* were placed in distinct clusters, with *Y. gloriosa* showing a pattern of mixed ancestry. The STRUCTURE analysis utilizing the USEPOPINFO flag indicated that alleles sampled in *Y. gloriosa* samples were shared with both parents with an average of 53% coming from *Y. aloifolia* (range: 43–66%) and 47% from *Y. filamentosa* (range: 33–57%)(Fig. 3b). Using the maximum-likelihood approach implemented in HINDEX, the mean hybrid index for all *Y. gloriosa* individuals was estimated to be 0.57 (standard error [SE] ± 0.074), suggesting that the nuclear genome of *Y. gloriosa* is approximately 57% *Y. aloifolia*-like and 43% *Y. filamentosa*-like (Fig. 4).

## Discussion

When taken together, both the life history data and the genetic data clearly support the intersectional hybrid origin of *Y. gloriosa*. In agreement with the morphological distinctness of *Y. gloriosa* and its hypothesized parental species (Trelease 1902), the PCO plot reveals three distinct clusters representing *Y. aloifolia*, *Y. filamentosa*, and *Y. gloriosa*. Furthermore, both Bayesian and maximum-likelihood methods confirm that the nuclear genome of *Y. gloriosa* is a mosaic of the hypothesized parental genomes. Based on data from two informative chloroplast loci, the plastid genome of *Y. gloriosa* was inherited from *Y. aloifolia*. Across all 14 nuclear loci examined, the parental species share only a single allele, likely as a retained ancestral polymorphism. This suggests that there is little to no introgression occurring between the hybrid and its parents. Additionally, sampled *Y. gloriosa* individuals display a wide range of genotypes at each locus including homozygosity for aloifolia-like or filamentosa-like alleles. The segregation pattern for alleles in the hybrid suggests that *Y. gloriosa* individuals are interbreeding to produce later-generation hybrids.

Of currently described homoploid hybrid species, the most common mechanism for isolating hybrid and parental populations seems to be habitat divergence (Gross and Rieseberg 2005). Ecological divergence may minimize both competition and interbreeding between hybrid and closely related parental populations. Transgressive segregation of parental traits may promote development of extreme traits in hybrid populations that allow them to thrive in new environments. For example, *Helianthus annuus* and *H. petiolaris* produced three hybrid species that exhibit divergent and extreme habitat preferences. Although *H. annuus* and *H. petiolaris* prefer mesic, clay-based soils and dry, sandy soils, respectively, their progeny prefer active sand dunes (*H. anomalus*), xeric habitats (*H. deserticola*), and desert salt marshes (*H. paradoxus*) (Rosenthal et al. 2002). *Pinus yunnanensis* and *P. tabulaeformis* hybridize to form *P. densata*, which inhabits extreme alpine environments. In contrast, the homoploid hybrid *Iris nelsonii* inhabits ecologically intermediate environments relative to its parental species. The hybrid *I. nelsonii* is found at intermediate water depths in cypress swamps, whereas *I. hexagona* thrives in open, deeper water and *I. fulva* inhabits shallower water in the understory.

Homoploid hybrid species rarely remain in local sympatry with its parental species. In 14 of 19 examples reviewed by Gross and Rieseberg (2005), habitat (vs., e.g., mating system) was the most important component of ecological divergence between hybrid and parental populations. Notable exceptions include the homoploid hybrid *Penstemon clevelandii*, which occurs in sympatry with its parental species, but is reproductively isolated due to a pollinator shift (Wolfe et al. 1998) and *Senecio eboracensis*, a tetraploid hybrid that is reproductively isolated from its tetraploid parent due in part to a shift in flowering phenology (Lowe and Abbott 2004).

It has been posited that the creation of a “hybrid habitat” through human-mediated or natural disturbance may promote the establishment of hybrid species (Anderson 1949). Dune habitats, where *Y. gloriosa* grows with *Y. aloifolia*, are dynamic with a high frequency of natural disturbance. Like *Y. aloifolia*, *Y. gloriosa* is able to propagate clonally through rhizomes and severed leaf tissue. This may contribute to the persistence of these species in disturbance-prone dune habitats. Both species (along with *Y. filamentosa*) also share the same moth pollinator, *T. yuccasella*. While all three species are known to flower simultaneously at some low frequency, their flowering times are largely nonoverlapping, with *Y. filamentosa* flowering the earliest and *Y. gloriosa* flowering the latest on average (Trelease 1893). *Yucca gloriosa*, therefore, joins a small list of homoploid hybrid species that has persisted in sympatry with one or both of its parental taxa.

Recent reviews on hybrid speciation (Chapman and Burke 2007; Paun et al. 2009) have found that the probability of polyploid (vs. homoploid) hybrid speciation increases with genomic divergence between parental species. At first glance, *Y. gloriosa* may seem to depart significantly from this pattern. The parental species are placed in reciprocally monophyletic sections of *Yucca* that have been separated by approximately 6.5 million years (Smith et al. #b^508^). Nonetheless, *Y. gloriosa* is a homoploid hybrid species. An analysis of 11.4 kilobases of chloroplast sequence data show a strikingly low amount of sequence divergence between *Y. aloifolia* and *Y. filamentosa* (uncorrected *p* distance of 1.776 × 10^−4^), suggesting that genetic distance is a more important impediment to homoploid hybrid speciation than phylogenetic (topological) distance. The paucity of genetic diversity within *Y. aloifolia* makes it impossible to determine with certainty whether *Y. gloriosa* is the result of a single or multiple hybridization events. Although only the *Y. aloifolia* chloroplast haplotype was observed in *Y. gloriosa*, the lack of intraspecific variation within *Y. gloriosa* makes it impossible to rule out multiple origins of the hybrid with *Y. aloifolia* serving as the maternal parent in each event.

The hypothesized hybrid origin of *Y. gloriosa* may be promoting diversification in associated yucca moths through host race formation. Host races have been described for the flowering stalk feeding “bogus” yucca moth species, *Prodoxus quinquepunctellus* (Svensson et al. #b^509^) and *P. decipiens* (Groman and Pellmyr 2000). Host race formation in *P. decipiens* occurred within the last 500 years following a host shift from *Y. filamentosa* to *Y. aloifolia* after the introduction of *Y. aloifolia* to the southeastern coast of the United States (Groman and Pellmyr 2000). Over a short period of time, host-specific *P. decipiens* populations have accumulated genetic, morphological, and phenological differences relative to each other (Groman and Pellmyr 2000). *Yucca gloriosa* represents another potentially even younger host for *P. decipiens*. Similarly, the divergence of *Y. brevifolia* into distinct subspecies is thought to have spurred the divergence of its pollinating yucca moth into species that display some degree of host specificity and reproductive isolation (Pellmyr and Seagraves 2003; Smith et al. 2009). Although *T. yuccasella* (the pollinator of southeastern United States yucca species) tends to be more of a generalist than other pollinating yucca moths, certainly the potential for host race formation exists.

## Conclusions

Hybrid speciation involving polyploidy has long been recognized as an important phenomenon in plant evolution (Soltis and Soltis 2009). Such events can create an instant barrier to reproduction with the parental species and may promote increased species and gene diversity. Furthermore, it is becoming increasingly clear that all angiosperms contain a polyploidization event in their evolutionary history (Goldblatt 1980; Lewis 1980; Masterson 1994; Soltis et al. 2009; Jiao et al. 2011). The impact of homoploid hybridization on biodiversity is less certain because backcrossing with parental species is often possible, blurring species boundaries. Indeed, this form of hybrid speciation can be difficult to detect and a small (but growing) number of examples exist in the literature (Gross and Rieseberg 2005).

The data presented here provide strong support for the hybrid origin of *Y. gloriosa* as the result of pollen dispersal from *Y. filamentosa* to the maternal parent, *Y. aloifolia*. *Yucca gloriosa* appears to be a later-generation hybrid that is reproductively isolated from its parents, likely due to differences in flowering phenology. Although more data are needed to assess whether *Y. gloriosa* is the product of one or more hybridization events, the data provided highlight the significance of this species as being the first genetically characterized homoploid hybrid yucca species between the monophyletic sections of Yucca and Chaenocarpa.
